# Research on Wavefront Sensing Applications Based on Photonic Lanterns

**DOI:** 10.3390/s25237300

**Published:** 2025-12-01

**Authors:** Zhengkang Zhao, Hangyu Zheng, Lianghua Xie, Jie Zhang, Zhuoyun Feng, Kaige Liu, Bin Zhu, Deen Wang, Ju Wang, Wei Liu, Qiang Yuan

**Affiliations:** Laser Fusion Research Center, China Academy of Engineering Physics, Mianyang 621900, China

**Keywords:** photonic lantern, wavefront sensing, adaptive optics (AO), focal-plane sensor, fiber optics

## Abstract

The Photonic Lantern (PL) is a novel fiber optic device emerging in wavefront sensing, which converts multimode fiber light fields into single-mode fields. By decomposing complex multimode fields into simple fundamental modes, the PL maps wavefront aberrations to light intensity. The Photonic Lantern Wavefront Sensor (PLWFS) functions as an ideal focal-plane sensor. It aligns the focal and imaging planes to coincide completely. This configuration mitigates Non-Common Path Aberrations (NCPAs), which traditional sensors struggle to resolve. This paper reviews the research history of the PLWFS. It first introduces the fabrication methods for PL, then focuses on illustrating the theoretical and experimental developments of the PLWFS. PLWFS research began with the initial realization of sensing simple tip/tilt aberrations, moved to establishing linear response models for small aberrations, and subsequently introduced methods such as neural network algorithms and broadband polychromatic light sources to achieve large aberration sensing and correction. This paper highlights significant research achievements from each stage, summarizes the current limitations in the research, and finally discusses the future potential of the PLWFS as an excellent focal-plane wavefront sensor.

## 1. Introduction

Adaptive Optics (AO) senses and compensates for wavefront distortions in the spatio-temporal domain. The concept was first proposed in the 1950s by Horace Babcock at the Hale Observatory to correct the atmospheric turbulence in stellar imaging [1]. Today, the application of AO has expanded far beyond astronomy, playing a crucial role in vital fields such as biological microscopy [2], laser communications [3], and industrial optical inspection.

The correction performance of an AO system often relies on the precision of the wavefront sensing. In practice, various types of wavefront sensors were developed, based on different principles. The most commonly used is the Shack–Hartmann sensor, which utilizes a microlens array to divide the incident wavefront into multiple sub-apertures. Each sub-aperture is focused into a spot, and by calculating the displacement of this spot from the optical axis, the slope of the sub-wavefront can be determined. The original wavefront phase is then reconstructed through an algorithm [4]. Besides the Shack–Hartmann sensor, there are other common wavefront sensing technologies including shearing interferometers [5] and curvature sensors [6]. The choice of wavefront sensing technology often depends on the specific application scenario.

As application scenarios expanded, traditional wavefront sensing methods could be limited sometimes. Traditional sensors like the Shack–Hartmann often require a separate sensing path created by beam splitters. Consequently, the sensing and imaging planes diverge, causing Non-Common Path Aberrations (NCPA) [7]. The NCPA is significant in atmospheric observations, especially for the sub-nanometer imaging applications. Furthermore, traditional imaging is insensitive to certain aberration modes, such as the discontinuous phase aberrations caused by pupil fragmentation, also known as “low-wind effect” (LWE) modes [8]. Researchers urgently seek superior focal-plane wavefront sensing solutions to address these challenges [9].

**Figure 1:**
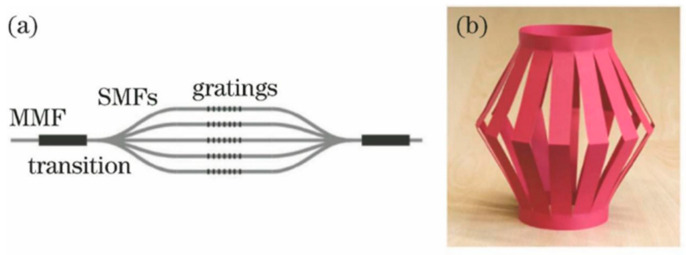
(**a**) The original PL concept [20]. (**b**) A paper lantern illustrating the structure [11].

The photonic lantern (PL) is a novel fiber-based optical device, the structure of which is illustrated in Figure 1. Its core function is to advance the light field coupling between single-mode and multimode [10]. This unique multimode to single-mode conversion mechanism combines a high optic collection efficiency of multimode fibers with a low-loss performance of single-mode fibers. The concept was first proposed by Birks in 2005, originally aimed at suppressing hydroxyl (OH) emission lines in the infrared band through a design that utilizes multimode fibers for collection and single-mode fibers for filtering [11].

With technological advancements, the application scope of PLs has expanded far beyond spectral filtering. In exoplanet detection, PLs have been successfully employed to eliminate mode noise, thereby facilitating high-precision radial velocity measurements [12]. Furthermore, the mode conversion capability of PLs has significantly advanced data transmission capacity in optical communications [13]. Additionally, these devices play a pivotal role in emerging fields such as distributed telescope synthesis [14].

In recent years, researchers have discovered that by leveraging the mode-coupling capability of the PL, a mapping relationship can be established between wavefront aberrations and the light intensity at the single-mode array ports [15]. This allows for the retrieval of wavefront distortions directly from the intensity distribution. The novel Photonic Lantern Wavefront Sensor (PLWFS) addresses several critical challenges inherent to traditional wavefront sensing technologies. By ensuring that the sensing plane coincides with the imaging plane, the PLWFS fundamentally mitigates Non-Common Path Aberrations (NCPA), a prevalent issue in conventional sensor systems [7]. Traditional sensors, such as the Shack–Hartmann, rely on dividing the wavefront into sub-apertures and are consequently insensitive to discontinuous phase steps [16]. Conversely, the PLWFS operates on the principle of mode coupling, encoding global phase information into the coupling of optical modes, which provides a unique capability to sense discontinuous aberration patterns. Furthermore, as an all-fiber device, the PL can function simultaneously as a sensing element and a feed for downstream instrumentation (e.g., spectrometers), thereby significantly reducing system complexity [13,17]. Finally, when integrated with advanced techniques such as neural networks, the PLWFS is capable of handling large-amplitude aberrations, demonstrating immense potential for applications in astronomical observation and complex wavefront correction [15,18,19].

## 2. Fabrication of Photonic Lanterns

**Figure 2:**
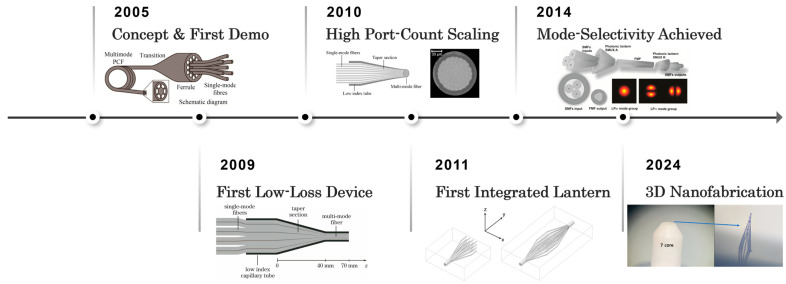
Development roadmap of PL fabrication technologies [13,20,21,22,23,24].

The PL originated from the need in astronomy to filter out unwanted light. In astronomical observations, hydroxyl (OH) emission lines from the atmosphere can interfere with measurement results, necessitating optical devices capable of filtering specific wavelength bands [20]. To validate this concept, Leon-Saval, Birks, and colleagues fabricated the first experimental PL [20]. The development roadmap of these fabrication technologies is illustrated in Figure 2. The fabrication involved inserting 19 standard single-mode fibers into a pre-formed silica capillary ferrule. This assembly, acting as a preform, was drawn on a fiber drawing tower, a process typically used for manufacturing photonic crystal fibers. The tapering process fused the fibers together and reduced their size, creating a multimode fiber at the narrow end whose cladding was formed by the surrounding air.

Although capillary-drawn PLs validated the concept, they suffered high optical coupling loss. Light transmission efficiency was only ~3.4% (equivalent to a 14.7 dB loss). Researchers attributed this loss to the significant mismatch in the number of supported modes between the two ends of the device. Based on estimations from scanning electron microscope (SEM) images, the multimode end had a core diameter of approximately 34.5 µm and a numerical aperture (NA) of about 0.75, allowing it to support over 710 modes. In stark contrast, the single-mode array at the other end, consisting of only 19 single-mode fibers, could support just 19 modes. This severe mode-mismatch dramatically increased the loss of the PL.

To improve the transmission efficiency of PL devices while simultaneously reducing fabrication complexity, Noordegraaf et al. designed and fabricated the first all-glass PL [21]. For this method, the PL was a combination of seven processed single-mode fibers inserted into a low-refractive-index glass capillary. The assembly was then stretched to four times its original length using a fusion tapering technique, which caused the thinner end to form a solid glass structure. In this configuration, the glass capillary shell became a new cladding, in which the original claddings of the single-mode fibers merged to form the new core, and the original single-mode cores were drawn down to a size so small that their influence became negligible. This approach reduced manufacturing complexity and produced a compact, all-glass structure. The fabricated PL exhibited excellent transmission performance, with a multimode-to-single-mode transmission loss of 0.32 dB and a single-mode-to-multimode loss of 0.24 dB, achieving a transmission efficiency of over 90%.

The fusion tapering method using a glass capillary subsequently became the most prevalent technique for PL fabrication. Building upon this method, researchers quickly produced lanterns with higher port counts (61 ports) to meet the demand in astrophotonics for fiber-based devices supporting a greater number of optical modes [22].

The ferrule tapering method relies on a fiber drawing tower or a specialized taper rig. The manufacturing process involves complex physical transitions and typically requires high temperatures exceeding 1700 °C [25]. The performance of a PL is critically dependent on whether the adiabatic condition within the taper transition region is satisfied [26]. Throughout the tapering process, precise control over the elongation speed and temperature is essential to prevent the formation of interstitial voids caused by residual air in fiber gaps or the occurrence of abnormal structural deformations [21]. Furthermore, dopant diffusion induced by high temperatures constitutes a significant factor influencing the performance of the PL [11]. To achieve low-loss coupling, the rate of change along the axis of the entire taper transition region must be sufficiently gradual to prevent energy coupling into radiation modes. Developing physical models that describe the viscous flow of fused silica glass to form a solid, circular fiber core represents a crucial direction in PL research.

To further reduce the gap between single-mode ports and improve the compactness of PLs, the concept of an integrated PL was proposed. As early as 1996, Davis et al. proposed fabricating optical devices in bulk glass using ultraviolet photons [27]. In their experiments, they observed that high-silica glass exhibited a change in refractive index when irradiated with a sub-ultraviolet laser. This change was confined to the laser scanning region. Building on this, in 2011, Thomson et al. first proposed manufacturing a three-dimensional integrated PL using ultrafast laser inscription [23]. The team directly designed a PL structure in borosilicate glass using femtosecond laser pulses, creating a 16-port ultra-compact device. The resulting lantern had an insertion loss of 5.7 dB and a mode coupling loss of only 0.7 dB, validating the feasibility of integrated PLs.

In 2014, the field of space-division multiplexing (SDM) in optical communications recognized the potential of the PL to serve as both a mode multiplexer and a demultiplexer. Leon-Saval et al. designed an improved fabrication method for a mode-selective PL [13]. This method relied on the careful design of the core properties and refractive indices of the input fibers. It enables stable coupling of the fundamental mode from each input fiber to a specific multimode fiber. Consequently, deterministic mode multiplexing and demultiplexing are achieved.

In 2024, Dana et al. demonstrated the first micro-scale PL fabricated using 3D printing technology, with the device having a total length of only 300 µm [24]. This fabrication method utilizes two-photon polymerization printing, allowing the PL to be printed directly onto the surface of other optical components, such as optical fibers or chips. This capability dramatically reduced the failure rate associated with subsequent manufacturing steps like splicing and alignment. 3D printing technology endows the PL with immense design freedom, laying a solid foundation for future miniaturization and integration.

Although Ultrafast Laser Inscription (ULI) and 3D nanoprinting offer more design freedom, the implementation of these technologies introduces some distinct challenges. PLs fabricated via ULI typically exhibit higher transmission losses (approximately 0.1–0.7 dB/cm) [23,28], primarily attributed to material absorption and scattering induced by the laser processing. Furthermore, the refractive index contrast achievable through laser processing has a distinct upper limit, presenting challenges in fabricating the high-contrast waveguides required for high-density PLs [29]. Regarding 3D printing technology, reducing surface roughness at the micro-scale remains difficult, which can lead to significant scattering problems [19]. Moreover, the micro-structures generated by both techniques persistently face mechanical stability issues in practical device deployment [24].

## 3. Current Status of Photonic Lantern Wavefront Sensor Research

The principle of the PLWFS is to map the wavefront distortion of an incident light field to an N-dimensional intensity signal at the single-mode ports through its unique tapered design. An incident light field with different wavefront distortions enters the multimode fiber and is represented as a superposition of several known modes. The phase information of the distorted spatial modes selectively excites these modes, resulting in a characteristic intensity distribution at the single-mode ports. Photodetectors measure the N-dimensional intensity which is used for wavefront reconstruction.

### 3.1. Initial Explorations

**Figure 3:**
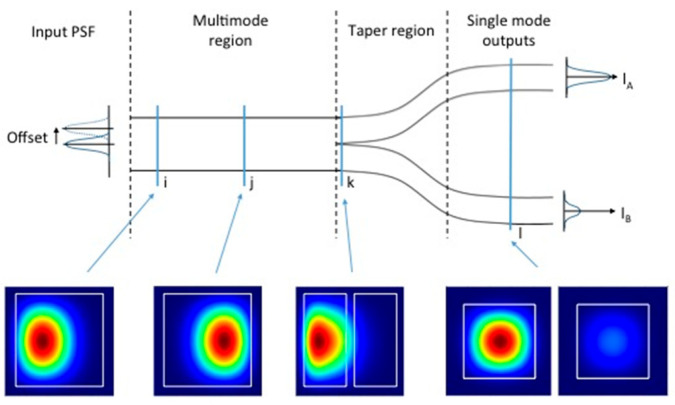
Effect of off-axis displacement on the intensity distribution in a PL [30].

In 2016, Corrigan et al. reported the wavefront coupling phenomenon from a focal plane to a simple four-mode PL. They analyzed the output intensity distribution of the single-mode cores when the input wavefront was affected by tilt, conceptually establishing a simple PLWFS [30]. As illustrated in Figure 3, by treating the output of each core in the PL as a quadrant in a quad-cell detector, they demonstrated that the offset of the input Point Spread Function (PSF) could be retrieved within a small linear range. The performance of the quad-cell and the PLWFS matched when the 1/e diameter of the PSF was less than 1/4 of the diameter of the PLWFS or the quad-cell.

**Figure 4:**
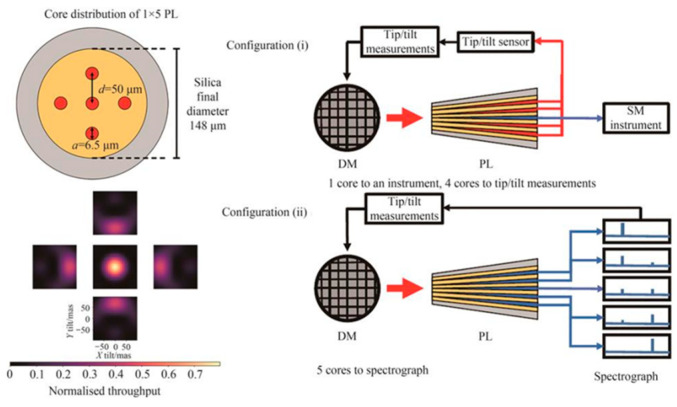
Two methods for sensing tip/tilt aberration with a 1 × 5 PL device [31].

Further, they designed and experimentally tested a 1 × 5 PL device, verifying its potential for wavefront sensing [31]. Figure 4 demonstrates the two configurations employed in this experiment for sensing tip/tilt aberrations. The experiment used a 5-port PL simultaneously as a low-order wavefront sensor and an instrument input fiber, co-locating the wavefront sensing plane with the focal plane to minimize non-common path aberrations. The PLWFS demonstrated a linear range of 55 milliarcseconds, which was larger than that of a quadrant detector (25 milliarcseconds). The advantage of the PLWFS lies in its dual functionality of wavefront sensing and light transmission, which can reduce the complexity of AO systems, while its large linear range is suitable for dynamic error correction.

Building on this, Cruz-Delgado et al. demonstrated a four-core multicore fiber PL tip/tilt wavefront sensor, obtaining the complex transmission matrix M from the isolated cores to the multimode fiber end-face, experimentally proving the feasibility of the PLWFS [32]. In this experiment, two receiver architectures were implemented to identify tip/tilt information. One PL imaged the four-core fiber face onto a 2D detector, and the other measured the power of the single-mode outputs by a multicore fiber multiplexer and photodetectors. For both schemes, an angular detection window of approximately 0.4° was achieved at 1064 nm.

In these studies, the PLWFS showed higher sensitivity than the Shack–Hartmann sensor, indicating its potential as a sensor for higher-order aberrations.

### 3.2. Theoretical Modeling

Early PLWFSs could only sense tip/tilt aberrations, and there was no suitable theory to describe their ability to sense higher-order aberrations. In 2022, Lin’s team developed a theoretical model for the aberration sensing process of a PL [33,34].

In the theoretical model, the entire response of the PL can be consolidated into a single complex transmission matrix A. The input electric field $u_{in}$ is transformed into the output electric field $u_{out}$ by the lantern, as described by Equation (1):

(1) $$u_{out}=Au_{in}$$

For a PL with N output ports and M input sampling points, the complex transmission matrix *A* is an N×M matrix.

Generally, photodetectors can only measure intensity information p, The output intensity $p_{out}$ is given by Equation (2):

(2) $$p_{out}={\left|u_{out}\right|}^{2}={\left|Au_{in}\right|}^{2}$$

This expression characterizes the entire process of wavefront sensing with a PL. The ultimate goal of the research is to invert this equation to recover the input light field from the measured intensity information.

For small phase perturbations, the input electric field can be expressed as:

(3) $$\begin{array}{l} u_{in}=U\exp(i\phi )\\ =U\exp(i\phi_{0})\cdot \exp\left[i(\phi -\phi_{0})\right]\\ \approx U\exp(i\phi_{0})\left[1+i△\phi \right] \end{array}$$

This expression holds when the phase perturbation △ϕ is very small.

From the expression, it can be seen that for small phase perturbations, the final output intensity will vary in a nearly linear fashion:

(4) $$p_{out}\approx {\left|A_{1}\right|}^{2}+B△\phi$$

The matrix *B* is called the phase interaction matrix of the PL, defined as:

(5) Bij≡2ImAij*∑kAik

where Im denotes taking the imaginary part, and A denotes the conjugate.

For low-order aberrations, the phase distortion can be expanded as a superposition of a modal basis (e.g., Zernike modes):

(6) △ϕ=Ra

where a is the vector of real-valued coefficients for the basis modes, and R is the basis transformation matrix.

Substituting this into the intensity expression, we get a linear response relationship for the intensity with respect to the basis modes:

(7) $$p_{out}\approx {\left|A_{1}\right|}^{2}+B^{\prime }a$$

where $B^{\prime }\equiv BR$.

By measuring the key matrix $B^{\prime }$, the input light field can be recovered by calculating the pseudo-inverse of $B^{\prime }$.

Lin used a numerical model based on the Python packages HCIPy and Lightbeam to simulate the entire process of PL wavefront sensing, demonstrating the theoretical performance of a 6-port PLWFS [33,34]. By numerically measuring the response matrix, the results showed that this configuration could sense wavefront aberrations from 0.25 to 0.5 radians RMS, with the maximum processable wavefront aberration being between 1 and 2 radians. In subsequent research, Lin discussed the factors affecting the sensing performance of the PL, analyzing the characteristics of different types of PLs. It was found that PLs with some degree of mode selectivity could handle a larger range of aberrations, while standard PLs without mode selectivity exhibited better linearity in their response. The paper also derived expressions for higher-order reconstruction models, but introducing higher-order models can lead to multiple solutions, which requires careful consideration in practical wavefront sensing applications.

In 2023, Lin’s team performed the first real-time test of a PLWFS’s performance in the near-infrared on the SCExAO testbed at the Subaru Telescope [35]. The device used in the experiment was a 19-port PL with a hexagonal array structure, fabricated using the tapering technique at the Sydney Astrophotonic Instrumentation Laboratory. When static aberrations within a range of 1.6 radians were injected, the PL was able to stably correct about 95% of the aberration. When dynamic aberrations varying on a 1 s timescale were introduced, the PLWFS was able to suppress the aberration to one-tenth of its original value. These results validated the feasibility of the PLWFS.

Lin and Fitzgerald (2024) proposed advanced nonlinear reconstruction techniques [36]. They introduced empirically calibrated phase retrieval methods, exploring two approaches to model the nonlinear intensity response: higher-order Taylor expansion and Radial Basis Function (RBF) interpolation. The higher-order Taylor expansion approximates the nonlinear regime in polynomial form, solving for the phase via the method of successive approximations. In contrast, RBF interpolation utilizes control points to construct either forward or backward models, establishing a mapping between light intensity and phase. Furthermore, they applied numerical continuation methods to address the intensity-to-phase inversion problem, providing a mathematical tool to characterize phase degeneracies and determine the invertible range of nonlinear sensors.

In 2025, the team incorporated spectral dispersion into the PL sensing methodology [37]. The research involved injecting a broadband light source into an N-port (19-port) PL; the output was then spectrally dispersed using a prism. This approach overcame the theoretical limit whereby a monochromatic PL can only sense N-1 modes (i.e., <19 modes). The authors posited that different wavelengths correspond to distinct projections of the wavefront aberration space, thereby providing additional dimensions of information for the linear model’s computations. This broadband wavelength dispersion technique (wavelength diversity) significantly enhanced the application potential of the PLWFS, laying the foundation for the future integration of combined wavefront sensing and spectrometer instrumentation.

Furthermore, Sengupta et al. (2025) investigated a critical engineering parameter, the injection f-number, to characterize the trade-off between the PLWFS’s sensing performance and its injection throughput efficiency [38]. The study assessed the sensing capability for the first 37 Zernike modes at different injection ratios, ranging from f/2 to f/12. Sacrificing throughput (f/8 to f/12) enhanced the PLWFS’s dynamic range for higher-order aberrations, specifically coma and trefoil. This study demonstrates that for applications specializing in sensing higher-order aberrations, it may be possible to enhance sensing capability at the expense of throughput efficiency.

These studies have typically treated the internal transmission process of the PL as a ‘black box,’ modeling the intensity-to-phase reconstruction solely from a mathematical perspective. If an accurate physical model of the PL can be obtained, this information can be utilized to significantly reduce the difficulty of wavefront reconstruction. Romer et al. employed a spatial light modulator (SLM) to generate a large number of complex input light fields, thereby exciting the modal fields at the multimode end of the PL [39]. By statistically analyzing the input conditions of the modal fields and the corresponding outputs from the single-mode array, the transmission matrix of the PL can be calculated. This matrix precisely describes how each orthogonal mode within the multimode fiber is mapped to the single-mode array.

Once this mapping relationship is determined, the input wavefront can be retrieved from the easily measurable output intensity data using an iterative algorithm. The research team adopted a limited-memory BFGS (L-BFGS) optimization algorithm, providing the Jacobian matrix and using a spectral method for initialization, which achieved rapid convergence of the algorithm [40]. In numerical simulation tests, the cosine similarity between the retrieved phase and the target phase was as high as 97%, laying a solid foundation for subsequent further optical applications.

While theoretical models provide the mathematical framework for phase retrieval in PLs, the structural optimization and design of these devices rely highly on numerical simulation. The Beam Propagation Method (BPM) is currently the most classic numerical simulation technique, which is capable of modeling the complete step-by-step evolution of light propagation. By simulating the propagation and evolution of the optical field within the nonuniform waveguide structure of the taper transition region, researchers can quantify the availability of the transition and evaluate mode-coupling loss.

Commercial software packages, such as RSoft and CUDOS MOF Utilities, are widely employed to optimize the design of PLs. These tools focus on the design of fiber cores and calculation of the photonic mode, which is crucial for the light propagation within the PLs [41].

### 3.3. Neural Network Technology

**Figure 5:**
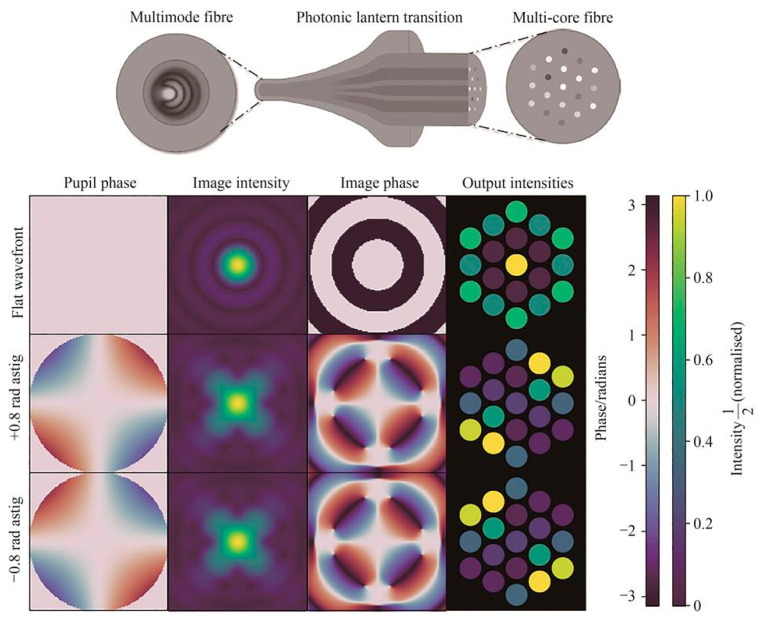
The response of a PL to aberrations with opposite signs, demonstrating its ability to break the sign ambiguity [15].

The linear response model of the PL is only an approximation that holds for small aberrations. For larger aberrations, the response is non-linear. To broaden the operational range of the PL and improve its sensing accuracy, Norris et al. introduced deep neural networks into the design of the PLWFS [15]. By using a neural network to learn the complex mapping between wavefront and intensity, the PL can reconstruct a wider range of wavefront aberrations without using linear approximations. Numerical simulation results showed that for incident light with the same intensity distribution, the PL could distinguish between positive and negative phases, as shown in Figure 5. In a 2020 laboratory demonstration, a PLWFS using a neural network was able to reconstruct the first 9 Zernike aberrations with a peak-to-valley (PV) value of around π radians, with a reconstruction accuracy of $2\times 10^{-2}\pi$ radians RMSE. The experiment also compared the neural network method with the traditional linear reconstruction method (SVD), proving that even with a small training set (about 480 samples), the error of the neural network method ($2\times 10^{-2}\pi$ radians) was better than that of linear reconstruction ($3\times 10^{-2}\pi$ radians). The team successfully reconstructed large-amplitude low-wind effect aberrations with a PV value of about 10 radians in the lab, far exceeding the linear range of the PL. Furthermore, in on-sky tests conducted on the SCExAO system at the Subaru Telescope, the PLWFS demonstrated a low-order aberration sensing capability comparable to that of a pyramid wavefront sensor [19].

In the following year, Sweeney utilized a neural network as a high-speed simulation tool, replacing the traditional beam propagation method (BPM) [18]. This approach increased the computation speed by more than five orders of magnitude while achieving a minimal mean squared error (MSE) on the test set, thereby demonstrating the effectiveness of neural networks as a simulation tool. By incorporating wavelength as a parameter in the study, the applicable range of the PLWFS was successfully extended from monochromatic light to broadband, polychromatic light scenarios, paving the way for further applications in astronomy.

In 2024, Wei et al. further explored the performance of the PLWFS under large-aberration, nonlinear regime conditions (RMS > 1.5 rad) [42]. The experiment focused on testing the PLWFS’s perceptual capability for the low-wind effect (LWE) and petalling modes. This validated the PL’s unique ability to sense these aberration modes, which are typically imperceptible to traditional wavefront sensors. The post-reconstruction RMSE for petal mode aberrations was only $2.87\times 10^{-2}$ rad, and even for the more complex low-wind effect, the RMSE was just $2.07\times 10^{-1}$ rad.

In the same year, Sengupta et al. compared the respective merits of three aberration reconstruction schemes: linear reconstruction, neural network (NN) technology, and the Gerchberg-Saxton (G-S) algorithm [43]. The simulation results demonstrated that the reconstruction accuracy of the NN technique surpassed the other two methods across all tested aberration amplitudes. This study confirmed the immense advantage of neural network technology in processing the inherently nonlinear signals from the PL’s output.

Limited by the fabrication process, the PL used in the demonstration could only support 19 modes, which restricted its response to higher-order aberrations. PLs capable of supporting more modes have already been successfully designed in the laboratory [11], and it is expected that higher-precision sensing of higher-order modes will be achieved in the future.

## 4. Conclusions

The PL’s unique multimode-to-single-mode coupling structure makes it an excellent focal-plane wavefront sensor. Research on the PLWFS is becoming increasingly advanced. Starting with simple tip/tilt aberrations, the sensing precision has been progressively improved. Through various approaches, including linear models and neural network algorithms, the performance of the PLWFS has been elevated to a level comparable to traditional wavefront sensors, and it even possesses unique advantages in sensing certain special modes. Concurrently, researchers are actively investigating novel approaches for utilizing PLs in wavefront sensing. Lu et al. (2024) proposed a method based on simultaneously measuring both the output intensity and the phase [44]. This approach acquires the complete output complex electric field vector, thereby obviating the need for nonlinear approximations or small-aberration assumptions. As a result, this method is capable of achieving exceptionally high reconstruction accuracy.

However, research on the PLWFS still has several limitations.

The study of wavefront sensing focuses on low-order aberrations, with research on high-order aberrations remaining unexplored and awaiting further investigation. The primary limitation restricting the capability of PLs to sense higher-order aberrations lies in the manufacturing process. While fabrication technologies for lanterns with 19 ports or fewer are approaching maturity, sensing higher-order aberrations necessitates scaling the device to hundreds of ports. In the fusion tapering process, the probability of manufacturing defects increases exponentially with the augmentation of the fiber count. Conversely, Ultrafast Laser Inscription (ULI) or 3D printing technologies struggle to minimize excessive scattering and mode crosstalk. Future research must prioritize automated, high-yield tapering protocols. Additionally, hybrid methodologies combining low-loss fiber tapering with additive manufacturing’s geometric flexibility are critical.

Traditional wavefront reconstruction algorithms are not perfectly matched to the physical characteristics of the PL, requiring the introduction of advanced algorithms like neural networks to improve wavefront sensing efficiency;

Currently, PLs are predominantly fabricated from fused silica, restricting their operational range to the near-infrared and visible bands. Extending the working bandwidth of PLs necessitates the processing of soft glasses, such as chalcogenide or fluoride glasses. These materials possess vastly different viscosity-temperature curves compared to silica and are prone to crystallization during the tapering process. Therefore, designing novel fusion tapering processes tailored to these materials is a prerequisite for fabricating the next generation of PLs.

This paper reviewed PL applications in wavefront sensing and discussed the PLWFS’s evolution. As scholarly inquiry advances, the PLWFS will be able to sense higher-order aberrations and is expected to advance the development of highly integrated optical systems.
